# Antimicrobial resistance in *Klebsiella* species from milk specimens submitted for bovine mastitis testing at the Wisconsin Veterinary Diagnostic Laboratory, 2008–2019

**DOI:** 10.3168/jdsc.2020-0031

**Published:** 2021-03-19

**Authors:** M.J. Fuenzalida, E. Furmaga, N. Aulik

**Affiliations:** 1 Division of Extension, University of Wisconsin, Madison 53706; 2 Wisconsin Veterinary Diagnostic Laboratory, University of Wisconsin, Madison 53706; 3 Department of Pathobiological Sciences, University of Wisconsin, Madison 53706

## Abstract

The purpose of this retrospective study was to describe antimicrobial resistance trends in *Klebsiella* isolates cultured from milk samples submitted to the Wisconsin Veterinary Diagnostic Laboratory for bovine mastitis testing. In vitro antimicrobial susceptibility testing was conducted in 483 *Klebsiella* isolates cultured from 63,841 milk samples submitted from January 1, 2008, through December 31, 2019. The Wisconsin Veterinary Diagnostic Laboratory conducted antimicrobial susceptibility testing according to the Clinical and Laboratory Standards Institute by using a broth microdilution panel. Ten antimicrobials were tested in the panel: ampicillin, penicillin, erythromycin, oxacillin + 2% NaCl, pirlimycin, penicillin/novobiocin, tetracycline, ceftiofur, cephalothin, and sulfadimethoxine. Isolates were considered resistant to specific antimicrobials based on minimum inhibitory concentrations described in Clinical and Laboratory Standards Institute guidelines. No guidelines were available for sulfadimethoxine; isolates were categorized as resistant when bacterial growth was not inhibited. The proportion of isolates resistant to ceftiofur, cephalothin, or tetracycline did not increase over time. For sulfadimethoxine, the proportion of resistant isolates decreased over time. These results do not demonstrate a trend toward increasing antimicrobial resistance among *Klebsiella* isolates. Despite that, antimicrobial resistance should continue to be monitored.

## Highlights

• *Klebsiella* isolates resistant to ceftiofur, cephalothin, or tetracycline did not increase.
• For sulfadimethoxine, the proportion of resistant isolates decreased over time.
• There was no trend toward increasing antimicrobial resistance among isolates.

## Graphical Abstract

**Figure:**
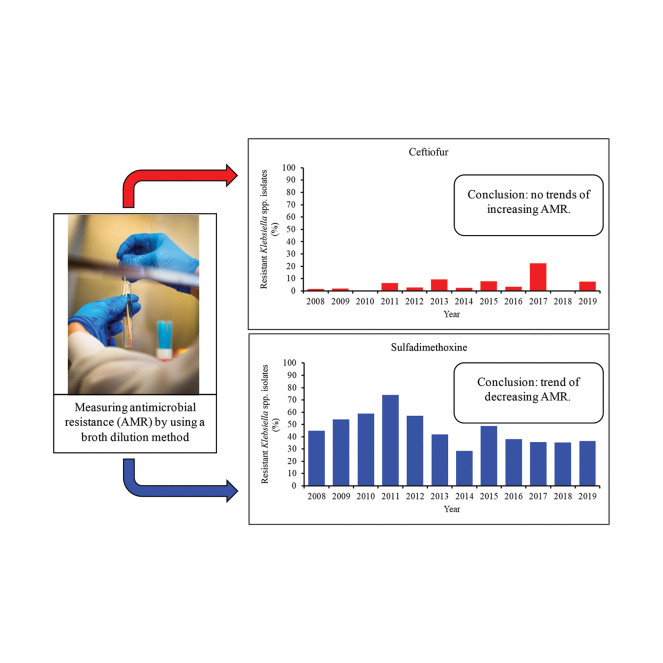
**Summary:** Mastitis is characterized as inflammation of the mammary gland caused by a variety of bacteria and is a costly disease for the dairy industry. *Klebsiella* species are important mastitis-causing pathogens not only because treatment with antimicrobials is unsuccessful, but also because their prevalence has been increasing over the years. It is important to monitor development of antimicrobial resistance in *Klebsiella* because there is a growing concern that it could negatively affect human health and it could be associated with poorer treatment outcomes in dairy cows.

Antibiotic resistance is a public health concern that calls for the judicious use of antibiotics in veterinary medicine. The US Food and Drug Administration has prohibited most extra-label uses of cephalosporin and fluoroquinolone drugs in the veterinary field to preserve these antibiotics for use in human medicine (FDA, 2018). Extra-label use of antimicrobials may represent a risk to public health because of its potential link with antimicrobial resistance (**AMR**).

The dairy industry is highly scrutinized due to usage of antimicrobial drugs in dairy cows for preventive or therapeutic purposes because consumers associate these practices with increasing AMR (Gulab, 2018). Mastitis is the most frequently diagnosed and treated disease in dairy cows. Mastitis is the inflammation of the mammary gland due to a bacterial infection and is classified as subclinical or clinical based on the absence or presence of clinical signs (abnormal milk, inflamed udder, sometimes accompanied by fever), respectively. Oliveira et al. (2013) characterized occurrence of clinical mastitis in 50 herds in Wisconsin and concluded that, in most cases, antimicrobial treatment decisions were not guided by etiology of the mastitis-causing pathogen. Despite the nonspecific use of antibiotics in the dairy industry, there is no evidence of AMR increasing over time among mastitis pathogens (Makovec and Ruegg, 2003; Erskine et al., 2004; Nóbrega et al., 2018).

A wide variety of bacteria can cause clinical mastitis, but environmental pathogens are most frequently isolated from cases of mastitis, especially *Escherichia coli* and *Klebsiella* species (Makovec and Ruegg, 2003; Oliveira et al., 2013). Most commonly in the *Klebsiella* genus, mastitis is caused by *Klebsiella pneumoniae* and *Klebsiella oxytoca* (Zadoks et al., 2011). In humans, *K. pneumoniae* is one of the main causes of infection in immunocompromised patients in hospital settings (Davis and Price, 2016). *Klebsiella* also causes one-third of opportunistic gram-negative infections, including wound, urinary tract, and nosocomial infections (Navon-Venezia et al., 2017). There is a growing concern that *Klebsiella* might be transmitted to people via consumption of raw milk and contaminated meat, although there is no evidence to substantiate that hypothesis (Davis and Price, 2016).

Surveillance of AMR development among *Klebsiella* species isolated from animals, especially dairy cows, is important because we need to investigate its potential effect on human health, if any, and we need to study the association between AMR development in *Klebsiella* species isolated from mastitis cases and treatment outcomes such as bacteriological cure (Fuenzalida and Ruegg, 2019), with a final objective of improving antimicrobial treatment decisions on dairies. The objective of this retrospective study was to describe AMR trends in *Klebsiella* isolates cultured from milk samples submitted to the Wisconsin Veterinary Diagnostic Laboratory (**WVDL**) for bovine mastitis testing between 2008 and 2019. We expected that AMR in *Klebsiella* species isolates would vary depending on the level of exposure of these bacteria to antimicrobials at the farm level; thus, ceftiofur resistance was expected to increase over the years, assuming that most cases of mastitis caused by gram-negative bacteria might be treated with ceftiofur formulations (Oliveira and Ruegg, 2014).

This was a retrospective longitudinal study. A total of 63,841 milk samples were submitted to the Madison and Barron locations of the WVDL between 2008 and 2019 for bovine mastitis microbiological testing. An accession is a single submission of one or more milks from a veterinarian from a single location. Milk samples from affected dairy cows submitted to the WVDL by a veterinarian were cultured using blood, eosin methylene blue, and TKT agar, as described in the National Mastitis Council Handbook (NMC, 2017) and approved by the American Association of Veterinary Laboratory Diagnosticians. *Klebsiella* was identified using biochemicals as described by the NMC Handbook (NMC, 2017) or matrix-assisted laser desorption/ionization time-of-flight (MALDI-TOF) described by the manufacturer (Bruker Daltonik). *Klebsiella* were identified to the species level when possible, which was more frequent when MALDI-TOF identification was used more consistently, starting in 2015. Therefore, all *Klebsiella* isolates, regardless of species, were used in this analysis. Of milk samples submitted, 1,694 (2.6%) *Klebsiella* species isolates were recovered from 63,841 individual milk samples. Of those, antimicrobial susceptibility testing (**AST**) was conducted in 483 *Klebsiella* species isolates. For financial reasons, the WVDL selected one *Klebsiella* species isolate from an accession regardless of the number of milk samples submitted in that accession unless directed by the submitting veterinarian to do otherwise. In vitro AST was conducted using the broth microdilution method (bovine mastitis panel, CMV1AMAF; ThermoFisher Scientific) and analyzed using the Sensititre platform (ThermoFisher Scientific) for all *Klebsiella* species isolates at the WVDL according to the Clinical and Laboratory Standards Institute (CLSI, 2018a, b). The antimicrobials included in this panel were ampicillin, ceftiofur (**CEF**), cephalothin (**CPL**), erythromycin, penicillin, penicillin-novobiocin, pirlimycin, oxacillin+2%NaCl, sulfadimethoxine (**SDM**), and tetracycline (**TET**). No MIC clinical breakpoints are available for bovine mastitis caused by *Klebsiella* species. Therefore, for CEF, MIC breakpoints from bovine mastitis *E. coli* were used (CLSI, 2018b), and for CPL and TET, MIC breakpoints from human *Enterobacteriaceae* were used (CLSI, 2020). Ceftiofur is considered a veterinary-specific antimicrobial, which should be considered for routine testing compared with other antimicrobials such as CPL and TET, which utilize breakpoints approved for human use of antimicrobials and should be selectively reported. Isolates were considered resistant to CEF and CPL when the MIC exceeded 4.0 μg/mL. Isolates were considered resistant to TET when the MIC exceeded 8.0 µg/mL. No MIC breakpoints are available for SDM (CLSI, 2018b); therefore, isolates were considered resistant when MIC exceeded 256 µg/mL. Other antimicrobials provided on the commercially available panel are either ineffective due to their spectrum of activity or *Klebsiella* species are intrinsically resistant (CLSI, 2018b).

Statistical analyses were conducted using SAS version 9.4 (SAS Institute, 2011). Descriptive statistics were performed using chi-squared or Fisher-exact analyses in PROC FREQ. Univariate associations between AMR and year were calculated using PROC GLIMMIX. The logistic regression model for the proportion of resistant isolates by year included resistance as a response variable (yes, no) and year as a continuous variable (0–11). To adjust for multiple *Klebsiella* species isolates obtained from single sources and included in the analysis, accession was included in the logistic regression model as a random factor. For all analyses, values of *P* < 0.05 were considered significant.

Based on microbiological results, 380 isolates (78.7%) were categorized as *Klebsiella* species, and 103 isolates were characterized at the species level; *Klebsiella oxytoca* (n = 23, 4.8%) and *Klebsiella pneumoniae* (n = 80, 16.6%). There was a statistical association between year and level of speciation (genus vs. species level, *P* < 0.001). Most identifications to the species level were made from 2014 to 2019 (n = 98, 95.1%), and the remaining 5 isolates were speciated in 2009 (n = 1), 2010 (n = 1), 2011 (n = 1), and 2012 (n = 2).

Susceptibility to CEF, CPL, and TET was tested consistently in 483 *Klebsiella* isolates. Sulfadimethoxine was the only antimicrobial in the bovine mastitis panel not tested in a few isolates due to unknown reasons. *Klebsiella* isolates were resistant to the remaining antimicrobials on the panel.

For CEF, 76.3 to 100% of *Klebsiella* isolates were susceptible at ≤0.5 or 1 μg/mL between 2008 and 2019 (Table 1). The proportion of *Klebsiella* isolates resistant to CEF was not linearly associated with year [odds ratio (**OR**) 1.13, 95% CI: 0.97–1.32; *P* = 0.106]; CEF resistance increased only between 2016 and 2017 (Figure 1). For CPL, 60.5 to 94.1% of *Klebsiella* isolates were susceptible at ≤2 or 4 μg/mL (Table 1). The proportion of *Klebsiella* isolates resistant to CPL was not linearly associated with year (OR 0.99, 95% CI: 0.91–1.07; *P* = 0.797), and no evident upward trend for resistance was observed (Figure 1). For TET, 54.2 to 92.3% of *Klebsiella* isolates were susceptible at 1 or 2 μg/mL (Table 2). The proportion of *Klebsiella* isolates resistant to TET was not linearly associated with year (OR 1.02, 95% CI: 0.95–1.09; *P* = 0.559), and no evident upward trend for resistance was observed (Figure 1). For SDM, 0.0 to 36.4% of *Klebsiella* isolates were susceptible at <32 or 64 µg/mL (Table 2). The proportion of *Klebsiella* isolates resistant to SDM was linearly associated with year (OR 0.93, 95% CI: 0.87–0.99; *P* = 0.017); there was a downward trend for resistance between 2008 and 2019 (Figure 1).

**Table 1:** Distribution [no. (%)] of *Klebsiella* species isolates, by ceftiofur and cephalothin MIC, recovered from milk samples submitted to the Wisconsin Veterinary Diagnostic Laboratory between 2008 and 2019 (n = 483)

Year	Total isolates (no.)	MIC distribution^1^									
		Ceftiofur (μg/mL)					Cephalothin (μg/mL)				
		≤0.5	1	2	4	>4	≤2	4	8	16	>16
2008	58	53 (91.4)	4 (6.9)	—	—	1 (1.7)	44 (75.9)	—	5 (8.6)	—	9 (15.5)
2009	50	41 (82.0)	8 (16.0)	—	—	1 (2.0)	39 (78.0)	5 (10.0)	1 (2.0)	1 (2.0)	4 (8.0)
2010	51	45 (88.2)	6 (11.8)	—	—	—	38 (74.5)	7 (13.7)	5 (9.8)	—	1 (2.0)
2011	31	24 (77.4)	4 (12.9)	1 (3.2)	—	2 (6.5)	18 (58.1)	9 (29.0)	1 (3.2)	—	3 (9.7)
2012	35	27 (77.1)	5 (14.3)	—	2 (5.7)	1 (2.9)	25 (71.4)	1 (2.9)	4 (11.4)	—	5 (14.3)
2013	43	30 (69.8)	6 (14.0)	2 (4.7)	1 (2.3)	4 (9.3)	23 (53.5)	3 (7.0)	1 (2.3)	2 (4.7)	14 (32.6)
2014	42	34 (81.0)	5 (11.9)	—	2 (4.8)	1 (2.4)	29 (69.0)	4 (9.5)	4 (9.5)	—	5 (11.9)
2015	38	27 (71.1)	8 (21.1)	—	—	3 (7.9)	25 (65.8)	7 (18.4)	1 (2.6)	1 (2.6)	4 (10.5)
2016	29	18 (62.1)	10 (34.5)	—	—	1 (3.4)	21 (72.4)	2 (6.9)	4 (13.8)	1 (3.4)	1 (3.4)
2017	76	44 (57.9)	14 (18.4)	1 (1.3)	—	17 (22.4)	44 (57.9)	11 (14.5)	3 (3.9)	—	18 (23.7)
2018	17	13 (76.5)	3 (17.6)	1 (5.9)	—	—	11 (64.7)	5 (29.4)	—	—	1 (5.9)
2019	13	12 (92.3)				1 (7.7)	7 (53.8)	5 (38.5)	—	—	1 (7.71)

1 The MIC breakpoints for ceftiofur were based on Clinical and Laboratory Standards Institute guidelines (CLSI, 2018b). *Klebsiella* isolates were considered resistant to ceftiofur or cephalothin when the MIC exceeded 4.0 μg/mL.

**Figure 1:**
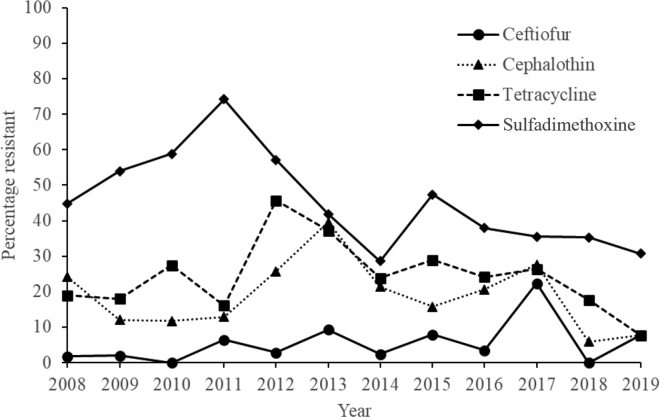
Percentage resistance to ceftiofur (n = 483), cephalothin (n = 483), tetracycline (n = 483), and sulfadimethoxine (n = 480) in *Klebsiella* species isolates during a 12-yr period. Percentage resistance was calculated as the number of isolates resistant to a specific antimicrobial divided by the total number of isolates tested for that antimicrobial.

**Table 2:** Distribution [no. (%)] of *Klebsiella* species isolates, by tetracycline and sulfadimethoxine MIC, recovered from milk samples submitted to the Wisconsin Veterinary Diagnostic Laboratory between 2008 and 2019 (n = 483)

Year	Total isolates (no.)	MIC distribution^1^									
		Tetracycline (μg/mL)^2^					Sulfadimethoxine (μg/mL)^3^				
		≤1	2	4	8	>8	≤32	64	128	256	>256
2008	58	25 (43.1)	21 (36.2)	1 (1.7)	—	11 (19.0)	1 (1.7)	5 (8.6)	16 (27.6)	10 (17.2)	26 (44.8)
2009	50	22 (44.0)	18 (36.0)	1 (2.0)	—	9 (18.0)	—	2 (4.0)	11 (22.0)	10 (20.0)	27 (54.0)
2010	51	9 (17.6)	26 (51.0)	2 (3.9)	—	14 (27.5)	—	—	2 (3.9)	19 (37.3)	30 (58.8)
2011	31	15 (48.4)	9 (29.0)	2 (6.5)	—	5 (16.1)	—	—	3 (9.7)	5 (16.1)	23 (74.2)
2012	35	13 (37.1)	6 (17.1)	—	—	16 (45.7)	—	1 (2.9)	2 (5.7)	12 (34.3)	20 (57.1)
2013	43	15 (34.9)	10 (23.3)	1 (2.3)	1 (2.3)	16 (37.2)	—	2 (4.7)	8 (18.6)	15 (34.9)	18 (41.9)
2014	42	20 (47.6)	12 (28.6)	—	—	10 (23.8)	—	2 (4.8)	8 (19.0)	20 (47.6)	12 (28.6)
2015^4^	38	16 (42.1)	9 (23.7)	2 (5.3)	—	11 (28.9)	—	—	3 (8.1)	16 (43.2)	18 (48.7)
2016	29	15 (51.7)	6 (20.7)	1 (3.4)	—	7 (24.1)	—	2 (7.0)	5 (17.2)	11 (37.9)	11 (37.9)
2017	76	44 (57.9)	12 (15.8)	—	—	20 (26.3)	—	1 (1.3)	6 (7.9)	42 (55.3)	27 (35.5)
2018	17	4 (23.5)	8 (47.1)	2 (11.8)	—	3 (17.6)	1 (5.9)	3 (17.6)	4 (23.6)	3 (17.6)	6 (35.3)
2019^5^	13	2 (15.4)	10 (76.9)	—	—	1 (7.7)	2 (18.2)	2 (18.2)	1 (9.1)	2 (18.2)	4 (36.4)

1 The MIC breakpoints for tetracycline were based on Clinical and Laboratory Standards Institute (CLSI) guidelines (CLSI, 2020).

2 *Klebsiella* isolates were considered resistant to tetracycline when the MIC exceeded 8.0 μg/mL.

3 CLSI does not provide breakpoints for sulfadimethoxine. *Klebsiella* isolates were considered resistant to sulfadimethoxine when MIC exceeded 256 μg/mL.

4 Sulfadimethoxine susceptibility testing was not conducted in 1 isolate.

5 Sulfadimethoxine susceptibility testing was not conducted in 2 isolates.

Results of this study indicate that the percentage of *Klebsiella* isolates categorized as resistant over the study years varied by antimicrobial tested. In fact, we observed decreasing trends in percentage of resistance among *Klebsiella* isolates for SDM. Contrary to our expectations, CEF resistance among *Klebsiella* isolates did not increase over the study years. The remaining antimicrobials tested for resistance did not increase during the study period.

Makovec and Ruegg (2003) described AMR patterns from a variety of mastitis-causing bacteria, including *Klebsiella*, and they concluded that there is no proof of increased AMR in bacteria isolated from milk samples for bovine mastitis testing sent to the WVDL between 1994 and 2001. As in the current study, they found a decreased AMR for trimethoprim-sulfamethoxazole (a compound similar to SDM); CEF was not included in their AST procedure. Erskine et al. (2002) described AMR patterns from mastitis-causing bacteria, including *Klebsiella*, isolated from milk samples sent to a laboratory between 1994 and 2000 and concluded there was no indication of increased AMR to antimicrobials that are commonly used in dairy cattle; for *Klebsiella* they did not observe upward trends in resistance against CEF. It is important to mention that differences in laboratory procedures for AST might influence how isolates are categorized as resistant or susceptible. Erskine et al. (2002) used the disk diffusion procedure and breakpoints based on National Committee for Clinical Laboratory Standards (NCCLS, now CLSI). Saini et al. (2012) described AMR for mastitis-causing pathogens isolated from different milk samples—from healthy udders and subclinical and clinical mastitis cases—collected on 89 dairy herds between 2007 and 2008; the AST procedure was similar to that used in this study. They found no increasing CEF resistance among *Klebsiella* isolates (total 139 isolates) collected from milk samples, which agrees with our results. Despite differences in laboratory procedures, our results agree with previous research conducted in similar (Erskine et al., 2002; Makovec and Ruegg, 2003) and different (Saini et al., 2012) sample populations in which there are no increasing trends in AMR in *Klebsiella* isolates.

Due to the nature of the data available for this study, some limitations need to be described. We could not obtain information about the type of mastitis (e.g., subclinical or clinical mastitis), clinical history, parity, or stage of lactation of cows from which milk samples were collected. When submitting to the WVDL, milk samples can only be submitted by herd veterinarians, who are not required to submit treatment history or other information about the herd. The only piece of data that we used to characterize herds from which samples were submitted was accession. We included accession as a random effect in our models to explain some variability due to milk samples that could have been sent from the same location over time. Limited information on plausible cow and herd risk factors might reduce the probability of explaining changes in AMR trends over time; in this study, year was the only explanatory variable used for statistical analyses. Decreasing numbers of milk samples submitted every year to the WVDL could affect the generalization of these results; thus, these findings need to be interpreted with caution. At the laboratory level, speciation was a limitation. At the WVDL, the majority of *Klebsiella* isolates were not speciated because it was more time consuming to do so before implementation of MALDI-TOF in 2015. Additionally, even when MALDI-TOF was available, most of the bacterial library consisted of human pathogens. No breakpoints for *Klebsiella* species causing mastitis in dairy cattle exist; therefore, breakpoints to categorize *Klebsiella* as resistant or susceptible are mostly available for bacteria isolated from human specimens. Research groups have described similar limitations when dealing with laboratory data (Erskine et al., 2002; Makovec and Ruegg, 2003; Saini et al., 2012). Using AST data is advantageous because these data are readily available, and it provides an opportunity for AMR surveillance in dairy cow populations and investigation of potential detrimental associations with human health.

Our results indicated no trends for increasing CEF resistance in *Klebsiella* isolates over time. It is important to mention that when we included accession as a random factor in our model, time went from being significant to not significant. Ceftiofur resistance remained consistently low between 2008 and 2016, spiked in 2017, and returned to low levels in 2019. A closer look at 2017 revealed that 55 of 76 isolates submitted to the WVDL belonged to the same accession (data not shown), explaining, in part, the spike. In addition to the source of *Klebsiella* isolates, other factors that could explain increasing CEF resistance during specific periods might be the treatment of mastitis cases using intramammary CEF formulations that could increase exposure of *Klebsiella* to CEF. A study of mastitis occurring in 51 dairy herds in Wisconsin noted that of the antimicrobial intramammary formulations used for treatment, CEF was the most frequently used, especially for gram-negative bacteria such as *Klebsiella* (Oliveira and Ruegg, 2014). On the other hand, odds of SDM resistance decreased over time, indicating that *Klebsiella* isolates used in this study might not have been exposed consistently to SDM. This might reflect the fact that farmers follow label specifications and use SDM only for dairy calves, heifers, and beef cattle. *Klebsiella* have the potential to acquire AMR genes from other bacteria and transfer plasmids (Nóbrega et al., 2013; Wyres and Holt, 2018). Despite the lack of evidence pointing to bovine *Klebsiella* mastitis pathogens resulting in human illness, β-lactams such as cephalosporins are frequently used in both veterinary and human medicine and it is therefore important to continue monitoring *Klebsiella* for ESBL (Nóbrega et al., 2013; Davis and Price, 2016). Further research is needed to build a more robust AMR database for veterinary medicine.

In conclusion, these results do not demonstrate a trend toward increasing AMR among *Klebsiella* isolates obtained from milk samples submitted to the WVDL. Antimicrobial resistance should continue to be monitored for surveillance purposes.
